# The role of trimethylamine-N-oxide level in the diagnosis of diabetic retinopathy and the differential diagnosis of diabetic and nondiabetic retinopathy

**DOI:** 10.5935/0004-2749.2021-0527

**Published:** 2022-10-19

**Authors:** Burkay Yakar, Erhan Onalan, Tugce Kaymaz, Emir Donder, Mehmet Ferit Gursu

**Affiliations:** 1 Department of Family Medicine, School of Medicine, Firat University, Elazig, Turkey; 2 Department of Internal Medicine, School of Medicine, Firat University Elazig, Turkey; 3 Department of Medical Biology, School of Medicine, Firat University Elazig, Turkey; 4 Department of Medical Biochemistry, School of Medicine, Firat University, Elazig, Turkey

**Keywords:** Biomarkers, Diabetic retinopathy, Differential diagnosis, Oxides, Type 2 diabetes mellitus, Trimethylamine-N-oxide, Trimethyloxamine, Biomarcadores, Retinopatia diabética, Diagnóstico diferencial, Óxidos, Diabetes mellitus tipo 2, N-óxido de trime-tilamina, Trimetilamina

## Abstract

**Purpose:**

Trimethylamine N-oxide serum levels have been associated with type 2 diabetes mellitus and its complications. The current study aimed to find out if plasma trimethylamine N-oxide level may be a novel marker in the diagnosis of diabetic retinopathy and if it can be used in the differential diagnosis of diabetic and nondiabetic retinopathy.

**Methods:**

The study included 30 patients with diabetic retinopathy, 30 patients with nondiabetic retinopathy, 30 patients with type 2 diabetes mellitus without retinopathy, and 30 healthy control participants. Biochemical parameters, serum IL-6, TNF-α, and trimethylamine N-oxide levels were measured in all participants.

**Results:**

Trimethylamine N-oxide level was significantly higher in diabetic retinopathy than in the other groups (p<0.001). There was no significant difference in trimethylamine N-oxide levels between nondiabetic retinopathy and control or type 2 diabetes mellitus Groups. There was a significant positive correlation between trimethylamine N-oxide level and elevated FPG, BMI, HOMA-IR score, BUN, IL-6, and TNF-α levels.

**Conclusion:**

The current study showed that the trimethylamine N-oxide level is elevated in diabetic retinopathy. These findings suggest that serum trimethylamine N-oxide level might be a novel marker for diabetic retinopathy, and it might be used in the differential diagnosis of diabetic and nondiabetic retinopathy.

## Abstract (pt)

**Objetivo:**

Os níveis séricos de N-óxido de trimetilamina têm sido associados ao diabetes mellitus tipo 2 e suas complicações. O presente estudo tem como objetivo responder a duas questões, entre elas: O nível plasmático de N-óxido de trimetilamina poderia ser um novo marcador no diagnóstico de retinopatia diabética? e Ele poderia ser utilizado no diagnóstico diferencial de retinopatia diabética e não diabética?

**Métodos:**

Trinta pacientes com retinopatia diabética, 30 pacientes com retinopatia não diabética, 30 pacientes com diabetes mellitus tipo 2 sem retinopatia e 30 participantes saudáveis do grupo controle foram incluídos no estudo. Parâmetros bioquímicos, níveis séricos de IL-6, de TNF-α e de N-óxido de trimetilamina foram medidos em todos os participantes.

**Resultados:**

O nível de N-óxido de trimetilamina foi significativamente maior na retinopatia diabética do que nos outros grupos (p<0,001). Não houve diferença significativa no nível de N-óxido de trimetilamina entre o grupo de retinopatia não diabética, do grupo controle ou do grupo de diabetes mellitus tipo 2. Houve uma correlação positiva significativa entre o nível de N-óxido de trimetilamina e os níveis elevados de FPG, IMC, HOMA-IR, BUN, IL-6 e TNF-α.

**Conclusão:**

O estudo atual mostrou que o nível de N-óxido de trimetilamina encontra-se elevado na retinopatia diabética. Esses achados sugerem que o nível sérico de N-óxido de trimetilamina pode ser um novo marcador na retinopatia diabética, podendo ser usado no diagnóstico diferencial de retinopatia diabética e não diabética.

## INTRODUCTION

Diabetic retinopathy (DR) is one of the most important and common end-organ injuries and complications of diabetes in middle-aged and elderly individuals. It is also an important public health problem due to a common visual impairment and vision loss. The prevalence of retinopathy among individuals with diabetes is estimated to be 34.6%. It has been reported that the incidence increases up to 90% in those with diabetes for more than 20 yr^(1)^.

In the pathogenesis of DR, oxidative stress caused by hyperglycemia is responsible for neurodegeneration. Vascular endothelial damage, disruption of the blood-brain barrier, vasoconstriction, capillary leak, and leakage of multiple inflammatory cytokines and plasma proteins can all be seen as the disease progresses. In the last stage, it is characterized by neovascularization and vitreous hemorrhage due to severe hypoxia. Although chronic hyperglycemia is held responsible for the development and progression of DR, the etiology has not been fully explained. Early diagnosis and treatment of DR are the most effective ways to delay the progression of the disease and prevent blindness, but in many cases, early diagnosis and effective treatment are insufficient. As a result, detecting new markers can be useful in DR management^(2,3)^.

Trimethylamine-N-oxide (TMAO), a microbiome-derived intestinal metabolite, has recently been suggested to be associated with various diseases. Many studies have reported a positive relationship between the level of TMAO concentration and the development of various diseases, such as cardiovascular diseases (CVDs) and cardiorenal disorders, including atherosclerosis, hypertension, ischemic stroke, atrial fibrillation, heart failure, acute myocardial infarction, chronic kidney disease, diabetes mellitus, metabolic syndrome, cancers (stomach, colon), as well as neurological disorders^(4,5,6,7)^. A recent me ta-analysis revealed a positive dose-dependent association between plasma TMAO and risk of diabetes^(8)^. Increased TMAO concentrations could impair glucose homeostasis, resulting in a worse clinical outcome of diabetic complications. Current data suggest that TMAO levels may be associated with diabetic complications. Considering the data presented above, the current study aimed to investigate whether plasma TMAO levels are associated with the presence of DRP in patients and compare them to patients with non-DR.

## METHODS

### Sample size and study population

This cross-sectional study was performed at the Department of Endocrinology and Ophthalmology between April and November 2021. Considering type I error (α) of 0.05, power (1-β) of 0.8, and effect size of 1.51, the minimum sample size required to detect a significant difference using this test that should be at least 11 in each group (44 in total). The study comprised a total of 120 participants. An endocrinologist diagnosed type 2 diabetes mellitus (T2DM) according to the American Diabetes Association guidelines. Participants aged 40-75 yr old were defined as eligible for the study. T2DM was diagnosed based on either the fasting plasma glucose (FPG) of ≥126 mg/dL (7.0 mmol/L) or 2-h PG ≥200 mg/dL (11.1 mmol/L) during oral glucose tolerance test or glycated hemoglobin (HbA1c) ≥6.5% (48 mmol/mol), or in a patient with classic symptoms of hyperglycemia or hyperglycemic crisis, a random plasma glucose >200 mg/dL (11.1 mmol/L)^(9)^.

The DR Group was defined as patients with T2DM and retinopathy. Initially, patients were evaluated by an endocrinologist for the diagnosis of T2DM, and the diagnosis of T2DM was confirmed by the endocrinologist. The diagnosis of DR was confirmed according to the international clinical DR and diabetic macular edema disease severity scales by two ophthalmologists(^10^). Non-DR groups were defined as, patients with retinopathy and without diabetes mellitus. The diagnosis of diabetes mellitus in patients was ruled out by the endocrinologist. Diagnosis of non-DR was confirmed by two ophthalmologists considering the absence of DR diagnostic criteria. T2DM group was defined as patients with T2DM and without any retinopathy. The diagnosis of T2DM was confirmed by endocrinologist, and two ophthalmologists confirmed no signs of retinopathy in patients. The control group consisted of healthy volunteers who did not report diabetes mellitus, retinopathy, and any chronic disease, as confirmed by endocrinologist and ophthalmologist.

Exclusion criteria included any other ocular diseases, pregnancy and lactation, cognitive impairments, autoimmune diseases, systemic infection, terminal illness, malignancy, another type of diabetes than T2DM, and steroid treatment and metabolic and endocrine diseases, which can affect glucose metabolism. This crosssectional study was approved by the Noninvasive Ethics Committee of the Firat University (date: 08.04.2021; no: 2021:05/41). The study was performed in accordance with the rules of the Declaration of Helsinki. Written consent was obtained from all participants in the study.

### Clinical data and biochemical analyses

The patients’ biochemical parameters were obtained from the university hospital database. Demographic characteristics, which included age, gender, and comorbid diseases, were examined. The standing height of patients were measured with 0.1 cm sensitive linear height scale. The weights of the patients were measured using a sensitive digital scale. Body mass index (BMI) was calculated using the conventional Quetelet formula (BMI=kg/m^2^). Two venous blood samples were collected after an overnight fast. One blood sample was sent to the clinical laboratory center of our hospital within 1 hr for further analyses of FPG, HbA1c, insulin, serum creatinine, and lipid profiles, including triglyceride, total cholesterol, low density lipoprotein cholesterol (LDL-C), vitamin D, alanine aminotransferase (AST), and aspartate aminotransferase (ALT) levels. For the IL-6, TNF-α, and TMAO analyses, a 5-ml blood sample was obtained. These samples were centrifuged at 5°C at 4,000 rpm and were stored at -80°C for further analyses.

### Analyses of the cytokine and TMAO

TNF-α and IL-6 concentrations were assayed by enzyme-linked immunosorbent assay (ELISA) kits (YL Biont, Shanghai, China) according to the manufacturer’s protocols. The intra-assay and inter-assay coefficients of variation are <10% and 12%, respectively.

TMAO concentration was performed in the Chemical Laboratory of Firat University by ELISA method. The concentrations of TMAO (human TMAO; catalog no: EA0141Hu; Bioassay Technology Laboratory, Shanghai, China), was measured using competitive ELISA kits obtained from Bioassay Technology Co., Ltd., Shanghai, China. The measurement range of the human TMAO kit was 150 to 10,000 ng/l and a sensitivity of 9.99 ng/L. The intra-assay coefficient of variation (CV) value is 10%, whereas the inter-assay CV value is 12%.

### Statistical analysis

Statistical analysis of the data was performed by IBM SPSS 22 statistics package program. The Shapiro-Wilk test was used to examine the distribution of continuous data. Descriptive data are given as mean ± SD for continuous variables with normal distribution, median (quartile 1-quartile 3) for continuous data with non-normal distribution, and number (*n*) and percentage (%) for categorical variables. For the comparison of more than two independent groups, we used the one-way ANOVA for normally distributed continuous data and the Kruskal-Wallis test for non-normal distributed continuous data. The categorical data was analyzed using Pearson chi-square test. The relationship between continuous variables was examined using Pearson correlation analysis or Spearman correlation analysis. A value of p<0.05 was considered statistically significant.

## RESULTS

A total of 120 participants (30 DR, 30 non-DR, 30 T2DM without retinopathy, and 30 control without diabetes mellitus and retinopathy) are included in the study. There was no significant difference of sex, AST, ALT, and VLDL levels among different goups. TNF-α level was significantly higher in both DR and non-DR than in the control. IL-6 level was higher in DR Group than in T2DM and control. TMAO level was significantly higher in DR than in other groups. There was no significant difference TMAO level between non-DR and control or T2DM groups. Demographic and metabolic characteristics are presented in table 1.

**Table 1:** Basic and metabolic characteristics of the study participants

	DRP	Non-DRP	T2DM	Control	*p* value
Age (yr)	61.3 ± 6.1*	59.5 ± 9.2	55.3 ± 10.2	42.9 ± 8.2	<0.001
Male	18 (60.0)	14 (46.7)	14 (46.7)	19 (63.3)	0.426
FPG mg/dl	201.8 (107.5)*+	92.3 (11.5)	184.2 (72.4)	85.5 (11.6)	<0.001
BMI (kg/m^2^)	28.2 (4.2)	27.2 (2.3)	29.2 (2.7)	26.5 (3.5)	0.009
HbA1c (%)	9.7 (2.4)*+	5.5 (0.5)	9.3 (3.4)	5.1 (0.6)	<0.001
HOMA-IR	6.6 (4.1-18.1)*+	1.5 (1.1-2.5)	6.0 (2.7-9.8)	1.9 (0.9-2.4)	<0.001
Triglyceride (mmol/L)	177.2 (85.0)*	135.3 (67.3)	164.1 (58.3)	128.6 (49.5)	0.015
TC (mmol/L)	187.7 (47.6)	174.5 (43.7)	193.8 (37.3)	184.7 (29.9)	0.312
LDL-C (mmol/L)	108.3 (36.1)	98.9 (28.9) ¶	128.1 (30.6)	106.8 (20.9)	0.002
VLDL	28.8 (21.0-60.0)	24.5 (17.5-36.5)	31.0 (23.0-41.2)	25.4 (18.4-40.0)	0.141
D vit	12.2 (6.7-16.3)*	14.7 (8.1-19.2)	17.8 (10.5-22.9)	23.4 (16.8-29.6)	<0.001
ALT	18.0 (14.0-26.3)	24.5 (17.8-30.0)	25.0 (17.5-30.5)	17.0 (14.0-29.0)	0.114
AST	17.0 (15.8-19.8)	25.5 (18.3-29.5)	19.0 (16.8-23.8)	19.0 (17.0-23.0)	0.145
Hb	12.9 (1.7)*	12.8 (2.3)	13.7 (1.8)	14.8 (1.2)	<0.001
PLT	272.0 (193-331)	216.0 (178-251)	248.0 (218.7-397)	238.5 (215-257.8)	0.049
BUN	48.5 (34.5-57.0)*α	43.0 (33.0-66.0)	32.5 (25.0-41.0)	30.5 (25.5-34.3)	<0.001
Creatine (mg/dl)	1.1 (0.4)*α	1.3 (0.6)	0.8 (0.2)	0.8 (0.1)	<0.001
IL-6 (pg/ml)	457.0 (295.8-560.8)*α	358.0 (331.5-416.3)	352.5 (270.0-389.8)	319.0 (269.0-410.3)	<0.002
TNF-α (pg/ml)	1,495.8 (1,272.9-2122.0)*	1,788.4 (1,629.3-3149.6)± ¶	1,284.7 (996.6-1,498.2)	1,049.1 (816.9-1,168.7)	<0.001
TMAO µmol/L	1,899.5 (914.5-2,515.0)*+α	757.7 (517.4-938.5)^a^	721.1 (538.2-1,215.3)^b^	300.0 (206.2-403.3)	<0.001

*p* values were obtained by one-way ANOVA or Kruskal-Wallis test for continuous variables and chi-square test for categorical variables. In post hoc pairwise comparison with Bonferroni correction.

Control= patients without diabetes and retinopathy; T2DM= diabetes without diabetic retinopathy; DRP= diabetic retinopathy; non-DRP= retinopathy without T2DM; BMI= body mass index; FPG= fasting plasma glucose; HbA1c= glycated hemoglobin; TC= total cholesterol; LDL-C= low density lipoprotein cholesterol; VLDL= very low density lipoprotein cholesterol; TMAO= trimethylamine-N-oxide.

* *p*<0.05 DRP compared with control.

+ *p*<0.05 DRP compared with non-DRP.

α *p*<0.05 DRP compared with T2DM.

¶ *p*<0.05 non-DRP compared with T2DM.

± *p*<0.05 non-DRP compared with control.

a *p*<0.05 non-DRP compared with control.

b *p*<0.05 T2DM compared with control.

**Figure 1:**
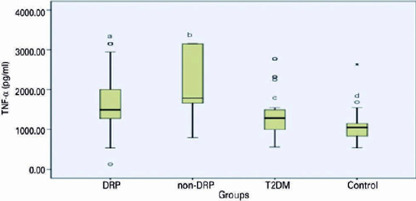
TNF-α levels and the association between groups. (TNF-α level was higher in DRP than control, p^a-b^: 0.001. TNF-α level was in non-DR than both T2DM and control. p^b-c^=<0.001 and p^b-d^=<0.001. DRP: diabetic retinopathy; non-DRP: non-diabetic retinophaty; T2DM: Type 2 diabetes mellitus without retinopathy).

**Figure 2:**
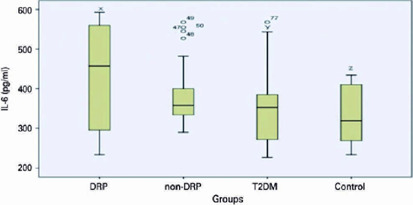
IL-6 levels and the association between groups (IL-6 level was higher in DRP than T2DM and control. Px-y: 0.003, Px-z: <0.001)

IL-6 level was higher in DR than in T2DM and control groups. There was no significant relationship in IL-6 levels between the DR and non-DR Groups. There was no significant difference in TNF-α level between DR and non-DR Goups. TNF-α level was higher in DR than in control and higher in non-DR than in both T2DM and control (Figures 1 and 2).

**Figure 3:**
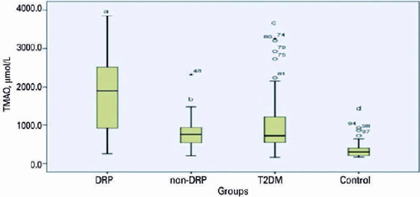
Plasma Trimethylamine-N-oxide (TMAO) levels (Plasma TMAO level was significant higher in DRP than other groups, multiple comparison p value: <0.001; binary comparison p values: a-b= <0.001; a-c= 0.001; a-d= <0.001. DRP: diabetic retinopathy; non-DRP: non-diabetic retinopathy; T2DM: Type 2 diabetes mellitus without retinopathy.)

Plasma TMAO level was higher in DR than other all groups (p<0.001). TMAO levels were significantly higher in the T2DM group compared with the control group (p=0.002). There was no significant difference between non-DR and control groups TMAO levels (p=0.284; Figure 3).

There was a significant positive correlation between TMAO level and age, FPG, BMI, HOMA-IR score, BUN, IL-6, and TNF-α levels. There was a significant negative correlation between TMAO level and vitamin D level (Table 2).

**Table 2:** Correlation between TMAO level and variables in all groups

Variable	TMAO µmol/L		Variable	TMAO µmol/L	
	r	p		r	p
Age/yr	0.386	**<0.001**	**D vit**	-0.228	**0.012**
FPG mg/dl	0.476	**<0.001**	**ALT**	0.045	0.628
BMI. kg/m^2^	0.220	**0.016**	**AST**	0.064	0.488
HbA1c. %	0.565	**<0.001**	**Hb**	-0.159	0.083
HOMA-IR	0.441	**<0.001**	**PLT**	-0.026	0.774
Triglyceride. mmol/L	0.114	0.214	**BUN**	0.320	**<0.001**
TC. mmol/L	0.073	0.430	**Creatine mg/dl**	0.177	0.053
LDL-C mmol/L	0.114	0.213	**IL-6 (pg/ml)**	0.305	**0.001**
VLDL	0.098	0.288	**TNF-**α **(pg/ml)**	0.230	**0.012**

r<0.2= no correlation; r= 0.2-0.4= weak correlation; r=0.4-0.6, a moderate correlation; r= 0.6-0.8, a strong correlation; *r*>0.8= a perfect correlation.

## DISCUSSION

DR is one of the leading causes of vision loss. It is emphasized that DR screening and early diagnosis are very important to reducing the negative effects of DR. During the pandemic period, patients have difficulty reaching a physician and restricting the close contact of the doctor, causing the patient to delay early diagnosis. The detection of novel biomarkers associated with DR may be helpful in early diagnosis and treatment. Based on this hypothesis, we sought for an answer to the question of whether serum TMAO level could be a novel marker for DR in the present study.

There is only one study in the literature that shows a relationship between TMAO and DR. In this study, Liu et al. reported that the TMAO level increased in DR^(11)^. However, this study has not investigated the relationship between non-DR and TMAO level. Our study showed that increased TMAO level is associated with DR, but there is no significant relationship between non-DR and TMAO level.

Many studies have reported a positive relationship between the level of TMAO concentration and the development of various diseases^(4,5,6,7)^. We focused on the effect of TMAO level on diabetic complications and vascular disease. Several studies have reported that higher TMAO levels are associated with adverse CVD outcomes in diabetic patients^(5,6)^. These findings suggest that TMAO levels may be associated with DR. While TMAO levels are high in DR, they are not associated with non-DR, which supports our hypothesis. Previous studies showed higher plasma levels of TMAO in T2DM patients than in nondiabetic patients. The current study showed that plasma TMAO levels increased in both T2DM and DR. Our study result agreed with previous studies. In addition, we showed that TMAO level was associated with DR and not associated with non-DR.

Recent studies support that increased plasma TMAO is associated with worse renal outcomes, impaired glycemic control, and increased diabetic complications^(12,13)^. Additionally, we observed a positive correlation between

TMAO and increased BUN, FPG, HbA1c levels, and HOMA-IR scores. In our study, there was no relationship between non-DR and TMAO. These data suggest that TMAO may be associated with diabetic complications, such as DR. Based on our data and the findings obtained from previous studies, it was believed that TMAO can be a new marker for DR^(11)^.

The current study had some limitations, including a relatively small number of patients from the same ethnicity and the study’s cross-sectional design. The study population might not be sufficient representative of the general population. Ignoring the stage of the disease in the DR patient group is another limitation of this study. Factors that may affect diabetic complications (duration of illness, medication use, compliance with treatment, etc.) could not be standardized. Finally, different lifestyle factors and comorbidities relevant to the disease were not considered, which might potentially influence the results.

In conclusion, high TMAO level is associated with DR and has no relationship with non-DR. A high TMAO level might be a risk factor for the development of diabetic complications, such as DR. Increased TMAO level may be due to hyperglycemia, insulin-resistant, and other diabetic complications, such as DR.

Our data indicate that future studies should focus on the effect of TMAO-lowering treatments on diabetes complications, such as DR. New studies with large populations are needed to clarify the role of TMAO in the diagnosis and treatment of DR.
